# Fungal Genomics in Respiratory Medicine: What, How and When?

**DOI:** 10.1007/s11046-021-00573-x

**Published:** 2021-09-07

**Authors:** Amelie P. Brackin, Sam J. Hemmings, Matthew C. Fisher, Johanna Rhodes

**Affiliations:** 1 MRC Centre for Global Disease Analysis, Imperial College London, London, UK; 2 Department of Infectious Disease Epidemiology, Imperial College London, London, UK

**Keywords:** Mycoses, Genomics, *Aspergillus fumigatus*, Respiratory diseases

## Abstract

Respiratory infections caused by fungal pathogens present a growing global health concern and are a major cause of death in immunocompromised patients. Worryingly, coronavirus disease-19 (COVID-19) resulting in acute respiratory distress syndrome has been shown to predispose some patients to airborne fungal co-infections. These include secondary pulmonary aspergillosis and mucormycosis. Aspergillosis is most commonly caused by the fungal pathogen *Aspergillus fumigatus* and primarily treated using the triazole drug group, however in recent years, this fungus has been rapidly gaining resistance against these antifungals. This is of serious clinical concern as multi-azole resistant forms of aspergillosis have a higher risk of mortality when compared against azole-susceptible infections. With the increasing numbers of COVID-19 and other classes of immunocompromised patients, early diagnosis of fungal infections is critical to ensuring patient survival. However, time-limited diagnosis is difficult to achieve with current culture-based methods. Advances within fungal genomics have enabled molecular diagnostic methods to become a fast, reproducible, and cost-effective alternative for diagnosis of respiratory fungal pathogens and detection of antifungal resistance. Here, we describe what techniques are currently available within molecular diagnostics, how they work and when they have been used.

## Introduction

The global burden of human associated mycoses is increasing. Each year, fungi account for billions of human infections and claim over 1.5 million lives worldwide [1, 2]. Of all reported fungal deaths, 90% are attributed to the genera *Cryptococcus, Candida, Histoplasma, Pneumocystis*, and *Aspergillus* [3]. Yet, up to 80% of patients could be prevented from dying, if fungal diagnostics were universally available and antifungal agents remain effective [1].

Advances in medical treatments and the increased use of immunosuppressive drugs has dramatically increased life expectancy. Consequently, the number of immunocompromised and immunosuppressed individuals, who are overwhelmingly susceptible to opportunistic fungal infections, has also increased. Individuals at risk from opportunistic fungal infections include those undergoing solid organ transplantation, haematopoietic stem cell transplantation and those with HIV/AIDS [4]. Moreover, patients diagnosed with chronic pulmonary disorders such as cystic fibrosis (CF) may undergo frequent hospitalisations due to cycles of lung inflammations from microbial colonizations and are at particular risk from opportunistic fungal infections. While bacterial colonizations are considered a primary concern, reports of yeasts and moulds in patient respiratory samples are increasing [5–7].

Although fungal pathogens can invade via any organ system, the primary route of infection into the body is via the respiratory tract [2]. Typically, in healthy individuals, conidia are quickly removed by mucocillary action, and those that are missed encounter neutrophils and alveolar macrophages [8], preventing attachment to the lung and subsequent infection. Continual exposure to microscopic fungi through daily inhalation of spores has the potential to cause a multitude of respiratory symptoms, which depending on the host’s immune response, can range from mild allergenic symptoms to life-threatening fungal invasions [2]. Such fungal invasions incur considerable economic burden on the health care system which is likely underestimated due to insensitive diagnostic techniques and lack of mycological surveillance [2].

The early diagnosis and implementation of effective antifungal therapy can benefit clinical outcomes [9–12]. However, the few classes of antifungals currently available are declining in efficacy due the emergence of drug-resistant fungal pathogens (Table 1). Continual exposure to antifungal compounds exerts selective pressures that can facilitate the evolution of resistance [13–16]. Additionally, fungi are equipped with intrinsic defence mechanisms, allowing them to overcome cellular stress and quickly adapt to tolerate hostile environments [17, 18]. Through adjusting cellular physiology and gaining beneficial mutations, fungi can thrive within the human lung, destroying crucial tissue, and invading vital organs. Research on the ecology, biochemistry, pathogenicity, and virulence of fungal infections is essential for the development of more efficient antifungal drugs. Yet fundamental challenges hamper the discovery of novel antifungal compounds that owe to the fundamental similarity in eukaryotic metabolism existing between fungi and humans [19] as well as a lack of fungal research initiatives and resource investment [20]. Considering this, preventative measures need to be implemented to reduce the risk of exposure to fungi in those most at risk from fungal infection. Several mycological surveillance studies have identified potential sources of contamination posing an increased risk for immunocompromised patients such as hospital construction, flower beds, and water systems [21–23].

**Table 1:** Four major classes of antifungal drugs and their mode of action

Class of antifungal	Antifungal drug	Mode of action	Susceptible fungi	Intrinsically resistant fungi	References
Polyenes	Amphotericin B	Binds to ergosterol in the cell wall causing changes to cell permeability	Mucorales	*Aspergillus terreus*	[81, 209–212, 216, 221]
			*Fusarium* spp.	*Scedosporium prolificans*	
			*Exophiala* spp.		
	Nystatin		*Aspergillus fumigatus*		[213, 214]
Azoles	Fluconazole	Disrupts the synthesis of ergosterol production via the inhibition of lanosterol 14- α demethylase		Mucorales	[208, 210, 211, 215, 226]
				*Fusarium* spp.	
				*Aspergillus fumigatus*	
	Posaconazole		Mucorales		[208, 211, 217, 220]
			*Aspergillus* spp.		
			*Exophiala* spp.		
	Voriconazole		*Scedosporium* spp.	Mucorales	[81, 211, 217, 220, 224]
			*Aspergillus* spp.		
			*Exophila* spp.		
	Itraconazole		*Aspergillus* spp.	*Fusarium* spp.	[215, 218, 220, 224, 226]
			*Exophiala* spp.		
	Isavuconazole		Mucorales		[211, 212, 216]
			*Aspergillus* spp.		
Echinocandins	Caspofungin	Disrupts the synthesis of β-glucan in the cell wall via the inhibition of 1,3-β-glucan synthase	*Aspergillus* spp.	*Cryptococcus neoformans*	[208, 209, 217, 221, 225, 226]
			*Pneumocyctis pneumonia*	Mucorales	
				*Fusarium* spp.	
	Anidulafungin		*Aspergillus spp.*	*Cryptococcus neoformans*	[208, 209, 223, 225, 226]
				Mucorales	
				*Fusarium* spp	
	Micafungin		*Aspergillus spp.*	*Cryptococcus neoformans*	[208, 209, 219, 225]
				Mucorales	
				*Fusarium* spp	
Antimetabolites	5-flurocytosine	Inhibits DNA and RNA synthesis	*Cryptococcus* spp.	*Fusarium* spp.	[222, 226]
			*Aspergillus spp.*		

This review focuses on the emergence of respiratory fungal infections with a particular focus on *A. fumigatus*. We highlight current and future molecular diagnostic tools for pathogen detection and surveillance. The rise in fungal respiratory infections and antifungal drug resistance mechanisms present significant challenges for health care systems. Among the respiratory diseases, *Aspergillus* infections are the most encountered. However, reports of non-*Aspergillus*, drug-resistant mould infections such as Mucormycosis*, Fusarium, Scedosporium, Exophiala*, and *Rasamsonia* are continuing to emerge in individuals at risk of fungal infections [24, 25]. Reports of fungal co-infection of the newly emerged cohort of high-risk COVID-19 patients are also of considerable clinical concern [26, 27]. Collectively, this means further research and routine surveillance is essential for understanding the epidemiology and clinical significance of emerging opportunistic fungal infections.

## Fungal Respiratory Infections

### *Aspergillus fumigatus*

**Fig. 1:**
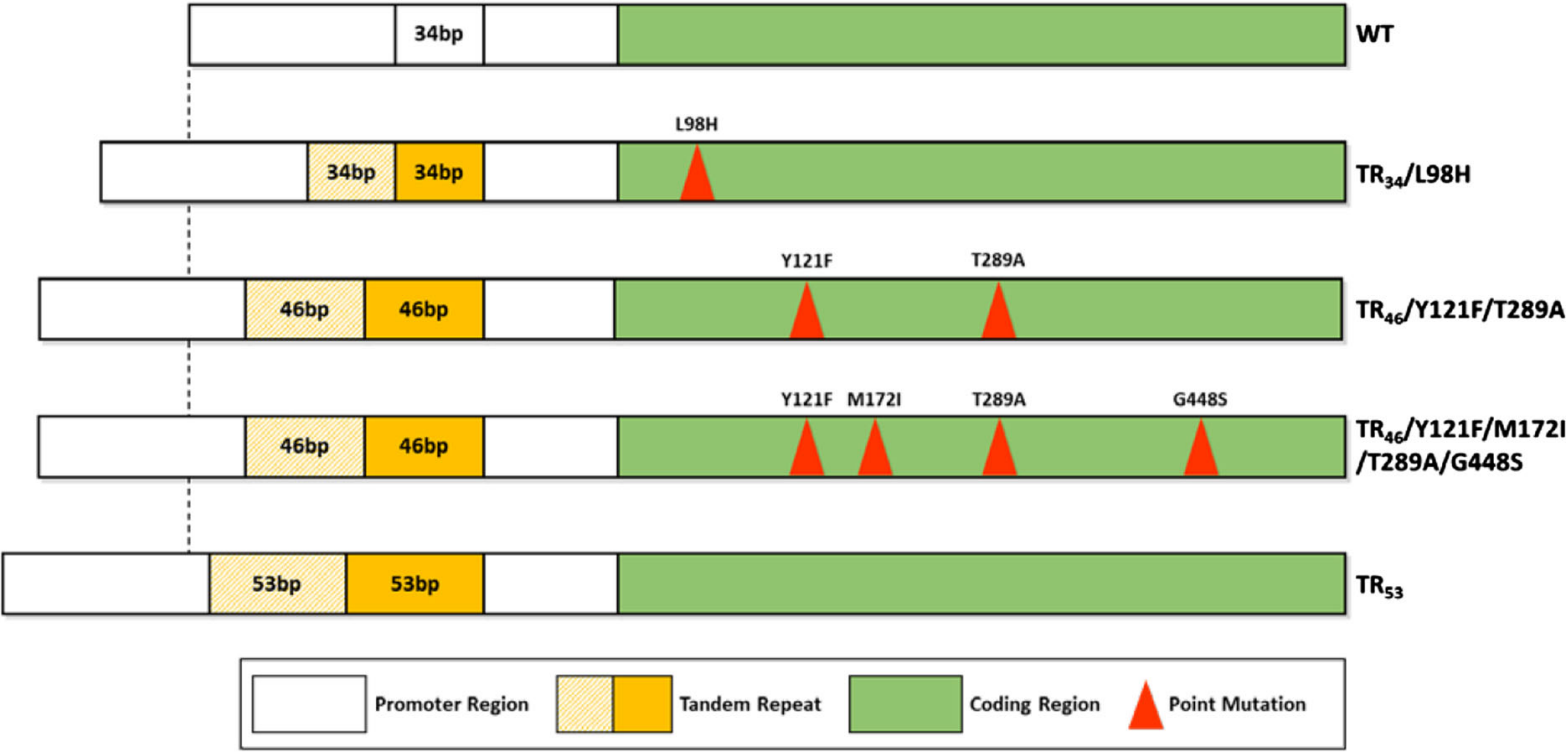
Illustration of common azole-resistance mutations that occur within the *cyp51A* gene within the promoter and coding regions (adapted from Zhang et al., 2017)

*Aspergillus fumigatus* is a leading cause of aspergillosis and one of the most frequently occurring eukaryotes on the planet. The ubiquitous global distribution of this species has been facilitated by the aerosolisation of conidia that are able to tolerate a wide range of biotic stresses [28, 29]. It is currently estimated that more than 3 million people suffer from chronic pulmonary aspergillosis (CPA) and around 300,000 people suffer from Invasive Aspergillosis (IA) of which there is an overall 50% mortality rate [2, 30]. IA is a leading cause of death in immunocompromised patients, particularly individuals with haematological cancers and transplant patients undergoing corticosteroid therapy [31]. Currently, there are only three classes of antifungals that are recommended for use to treat aspergillosis: these are the azoles, polyenes, and echinocandins. For treatment of invasive or chronic aspergillosis the primary treatment is using the azole voriconazole, while polyenes and echinocandins are recommended as salvage therapies if the patient’s infection becomes refractory or intolerant to the primary treatment [32, 33]. In recent years, *A. fumigatus* has been rapidly gaining resistance to the azole drug group. Typically, azole resistant *A. fumigatus* (AR*Af*) is synonymous with substitution of specific amino acids within the *cyp51A* gene and the insertion of tandem repeats in the promoter region that lead its overexpression (Fig. 1) [34]. This is of great concern as the mortality rate for individuals infected with resistant isolates exceeds 80% [35, 36]. With the emergence of azole resistant phenotypes, surveillance studies are key for understanding the overall burden of azole resistance in health care settings and the environment. Genomic analysis of *A. fumigatus* isolates collected during nosocomial studies have identified a high prevalence of azole resistance of ~ 16% in CF patients [37] and up to 16% in soil in central London [29]. Importantly, some patients with chronic respiratory disease have never been exposed to antifungal therapy yet are infected with *A. fumigatus* carrying pre-adapted antifungal resistance mutations, indicating an environmental route of transmission [38]. Resistant alleles TR_34_/L98H and TR_46_/Y121F/T289A have also been shown to appears on both clonal backgrounds in the environment and in clinics [39].

It has been observed that influenza-associated pulmonary aspergillosis complicates the clinical progression of many patients with acute respiratory distress syndrome (ARDS) [40–42]. The pulmonary epithelial damage caused by severe influenza and use of corticosteroids appear to be the main risk factors in developing IA co-infection [43, 44]. Several studies of co-infections during the H1N1 pandemic in 2009 and the Middle East Respiratory syndrome (MERS) outbreak in 2012 linked IA to higher mortality rates [42, 45, 46]. COVID-19 has been rapidly spreading across the world since late 2019, and to date, the number of confirmed cases exceeds 110 million people and has resulted in nearly 2.5 million deaths [47]. It has been shown that up to 40% of COVID-19 hospitalized patients can develop ARDS and are therefore potentially susceptible to co-infections caused by both bacteria and *Aspergillus* spp. [48–50]. Several reports of COVID-19 associated pulmonary aspergillosis (CAPA) have raised concerns that the fungal co-infection is a contributing factor to mortality [51–54] with estimates of mortality approximately 47–52% [55, 56]. Recently, published data from across Europe describe high incidence rates of 12–33% of CAPA in patients admitted to ICU with severe COVID-19 [51, 57–65] and is likely to be associated with the use of corticosteroid use as a contributing risk-factor [64, 66, 67]. Given the difficulties in routine screening; overburdened health care system, handling of Hazard Group 3 samples and lack of testing capabilities, the true estimates of CAPA are likely to be grossly underestimated [66, 68, 69]. Timely surveillance and further research into the impact of *Aspergillus* co-infections in COVID-19 is essential for improving diagnosis, treatments, prophylaxis, and understanding the relationship with host immunological responses [66, 68].

### *Scedosporidium*

After *Aspergillus*, *Scedosporium* ranks second as the most common fungi associated with chronic airway colonization in CF patients [70]. Molecular taxonomy has identified the most clinically relevant species as *Scedosporium apiospermum*, *Scedosporium boydii*, *Scedosporium aurantiacum*, *Scedosporium dehoogii* and *Scedosporium minutisporum* [71, 72]*.* Large variations in frequency in CF are often reported, partly due to difficulties in detection methods [73]*.* Positive diagnosis is largely reliant on the detection of fungal growth from clinical samples. However, the species complex is typically slow growing with an incubation period of up to 4 weeks and often out competed by high frequencies of rapidly growing fungi. [74]. The development of selective media such as SceSel + [74] and nucleic acid sequencing-based techniques [75] has vastly improved the detection of culture sensitive species. *Scedosporium* have shown resistance to a wide range of antifungals including polyenes (amphotericin B and nystatin) and azoles (fluconazole, itraconazole, and isavuconazole) and a reduced susceptibility to the echinocandins (caspofungin and anidulafungin) [76–81]. Furthermore, antifungal susceptibility varies between species which often makes treatment difficult [82, 83]. Species identification and susceptibility testing is highly recommended for effective treatment [81]. A draft whole genome sequence, which could be used as a reference genome, of *Scedosporium boydii* was only produced in 2017 [84]. Another reference genome was generated in 2020, this time of *Scedosporium apiospermum* [84]. While obviously a clinically relevant fungal pathogen, there is a lack of studies applying whole genome sequencing (WGS) to outbreaks of this disease, highlighting an urgent need. It is imperative that WGS is applied to other *Scedosporium* spp. in a bid to elucidate the mechanisms for antifungal tolerance.

### Mucormycosis

Mucormycosis is a rare, aggressive infection caused by the filamentous fungi of the order Mucorales and is associated with high rates of morbidity and mortality when found. Owing to the rarity of the disease, data regarding diagnosis, treatment and epidemiology are often lacking [85, 86]. The most frequent causal agents of mucormycosis include *Rhizopus* spp., *Lichtheimia* spp., *Apophysomyces* spp., *Rhizomucor* spp., *Mucor* spp. and *Cunninghamella* spp. [86, 87] and are commonly detected in patients with prolonged neutropenia and recipients of solid organ or haematopoietic stem cell transplants [88, 89]. Patients with diabetes mellitus, prolonged neutropenia and solid organ transplants are particularly at risk of developing mucormycosis infections [90]. Recently, outbreaks of mucormycosis have been reported in COVID-19 patients that have the associated risk factors diabetes mellitus and corticosteroid therapy, especially in the Indian subcontinent [27, 91–93]. Due to its rapid clinical progression, early diagnosis is essential for the implementation of therapeutic plans and prevention of angioinvasion and tissue necrosis [94]. However, diagnosis is limited by low culture sensitivity and often relies on the identification of hyphae in lung biopsies [90]. Furthermore, treatment can be difficult due to resistance to several of the available antifungals such as voriconazole and the echinocandins [95, 96]. Posaconazole and isavuconazole have shown in vitro efficacy but typically amphotericin B is recommended for first line therapy with possible surgical intervention [90, 97, 98]. Recently, whole genome sequencing (WGS) has been used effectively to resolve outbreaks of Mucorales spp. in hospitals. In 2019, an outbreak of mucormycosis was reported within heart and lung transplant patients over a 6 month period [99]; WGS was used to determine linkage of patients during traditional epidemiological investigations, surmising that the patients were unlikely to have been infected from a common source due to the disparate nature of the isolates. Similarly, a larger outbreak, this time of *M. circinelloides* f. *circinelloides* within a burns unit used WGS to resolve what appeared to be genotypically divergent isolates according to ITS sequencing [100]. WGS analysis provided additional insight into the nature of the outbreak, revealing that the patients were again not infected from a clonal strain, but from a pool of diverse strains in the environment. These analyses also concluded that direct transmission was also unlikely.

### Fusaria

The genus *Fusarium* harbours important plant pathogens that are known for their wide variety of infection mechanisms [101, 102]. Opportunistically acquired *Fusarium* are also responsible for causing a broad spectrum of respiratory diseases in immunocompromised hosts, including allergic sinusitis [103], chronic non-invasive sinusitis [104] and disseminated infections in severely immunocompromised patients. Nosocomial surveillance studies have recovered *Fusarium* species from hospital water systems, and hospital air investigations into the molecular relatedness between the patient and environmental isolates identified the dispersion of conidia through showering can lead to the transmission of airborne conidia to the immunocompromised host [23]. Treatment of fusariosis, in particular infections caused by *Fusarium solani*, is made difficult by intrinsic resistance to many antifungal drugs, including echinocandins [105] and azoles [106, 227]. The polyene antifungal, natamycin is active against *Fusarium* spp. and is often used in combination with voriconazole for *Fusarium*-associated fungal keratitis [105, 107]. Acquired resistance mechanisms, including efflux pump overexpression and *cyp51* overexpression have been linked to elevated resistance to agricultural fungicides in plant pathogenic *Fusarium* spp. [108] and phenotypes displaying cross-resistance to medical antifungals has been observed [109]. While WGS has been used to investigate the population structure and architecture of the *Fusarium* genome, this technology is yet to be applied (to the authors knowledge) to human infections. Given the large estimated global burden of *Fusarium* infections (approximately 1–1.2 million cases annually) [2], this is large public health concern which would benefit from applications of WGS, as demonstrated effectively in other clinical fungal infections.

### *Exophiala*

The melanized yeast-like fungus, *Exophiala dermatitidis*, is a globally distributed ascomycete fungus found naturally in soil and plant debris, but also in very humid environments such as kitchens and dishwashers, where it occurs on rubber seals assisted by the formation of biofilms [110]. Generally, incidence of infection in humans caused by *Exophiala* spp. are uncommon, however there has been an increase in the reporting of *E. dermatitidis* as a causative agent of human disease in both immunocompetent and immunocompromised hosts [111–115]. Infections that are caused by *E. dermatitidis* can be subdivided into three groups: superficial, cutaneous and subcutaneous, and systemic or visceral. Superficial infections often relate to surgical operation or trauma and non-superficial infections occur in patients with predisposing illness [116]. CF patients have displayed a high rate of *E. dermatidis* where it can be commonly found in respiratory tracts [117] and is thought to be pathogenic [118] Identification of *Exophiala* spp. is commonly established through the isolation of strains using either Sabouraud agar (SAB) or erythritol chloramphenicol agar (ECA), followed by sequencing of the fungal-specific ITS marker regions (ITS1, ITS2) [119]. Very little is known about the true susceptibility status of *Exophiala* spp. to antifungal agents, however these infections appear to be susceptible to a range of azoles including voriconazole, itraconazole, and posaconazole, but show reduced susceptibility to echinocandins and fluconazole [119, 120].

While the *Exophiala* species complex is large, a considerable number of studies have applied WGS to both environmental and clinical *Exophiala* isolates. Notably, a study produced in 2020 analysed both the genomes and transcriptomes of *E. dermatitidis* isolates, indicating that some strains of this species are able to alter their transcriptomes to cope with moderate stress, before returning to the original state [121]. This could provide a mechanism of adaptation to the human host. This was corroborated by an additional study using long read sequencing and transcriptomics, revealing adaptive strategies within *E. spinifera*. It is possible that *Exophiala* spp. are therefore able to adapt to habitat choice and cause infection in susceptible patient populations. Future studies employing WGS, in addition to transcriptomics, will be useful to explore these adaptive strategies and mechanisms of pathogenicity further [122].

### *Cryptococcus gattii*/*neoformans* species complexes

Pulmonary cryptococcosis is a lung infection that may cause acute pneumonia progressing to ARDS and, if dissemination occurs, fungal meningitis. The disease is caused by encapsulated yeasts *C. neoformans*/*gattii* that belong to the phylum Basidomycota. These yeasts are commonly found associated with trees, animals, birds, and their faeces and cause infection following inhalation. In the lower airways, pulmonary macrophages engulf and kill *Cryptococcus* however in some cases (often associated with risk factors such as HIV-AIDs), yeasts can survive to cause pulmonary infection, ARDS and onwards dissemination to the meninges [123]. Radiological diagnosis of pulmonary cryptococcosis is difficult as the inflammatory changes and nodules are similar to those associated with other infections including mycobacteria and viruses, as well as malignancies. Laboratory diagnosis of the infection traditionally relies on visualizing the characteristic encapsulated yeasts in respiratory samples or direct culture. However, detection of cryptococcal antigen (CrAg) using lateral flow tests has recently revolutionized the diagnosis of this disease in a variety of clinical settings.

The use of genomics has been key to our understanding of the epidemiology of cryptococcosis and has a substantial body of literature owing to the use of genomics in this area. The use of WGS from serially collected isolates of *C. neoformans* was integral for determining whether HIV/AIDS patients were undergoing recurrence of infection due to relapse of the original infecting isolate, or re-infection with a different isolate. The study by Rhodes et al*.* [124]*,* demonstrated that patients were most likely experiencing recurrent infections due to a relapse of the original infecting isolate, due to the clonal relationship and low genetic diversity between serially collected isolates. However, this study also utilized WGS to show that some isolates displaying high levels of genetic heterogeneity due to nonsense mutations within *MSH2,* the gene encoding DNA mismatch repair, leading to a hypermutator state, allowing evolution to antifungal drugs. Further, experimental studies have confirmed that the hypermutator state within the closely related *Cryptococcus deuterogattii* also allows within-host adaptation [125]; it is therefore plausible that the *C. neoformans* hypermutator state could similarly lead to within-host adaptation.

Later, studies using serially collected *C. neoformans* isolates have investigated the evolution to antifungal drugs further; a study by Stone et al. [126] used WGS to conclude that aneuploidy within *C. neoformans* drives heteroresistance to fluconazole-only drug therapy. This finding is clinically relevant and has real-world implications in the treatment of cryptococcosis, which encourages the use of combination drug therapy.

Genomics has also proved useful when investigating outbreak of *C. neoformans*; an outbreak in a Glaswegian hospital in 2018 was investigated with genomic epidemiology and found that isolates were genetically distinct. Genomic analyses were crucial to identifying that patients likely acquired the infection independently, rather than direct transmission of a clonal isolate [127].

### Pneumocystis pneumonia (PCP)

Pneumocystis pneumonia (PCP) is a serious respiratory infection caused by the yeast-like fungus *Pneumocystis jirovecii*. It is the major causative agent of a life-threatening pneumonia in immunosuppressed patients [128, 129]. Incidence of *P. jirovecii* infection in healthy people ranges between 0 and 20% [130]; although lung autopsies of individuals that died accidentally, followed by a nested PCR approach, found that an asymptomatic *P. jirovecii* pulmonary infection is prevalent in more than half of the general population [131].

*Pneumocystis jirovecii* infection becomes much more serious in patients with prior medical conditions that has weakened their immune system. Notably, the prevalence of PCP suddenly spiked in the 1980s in homosexual men in the USA, trigging the AIDS epidemic in San Francisco, prior to the identification of HIV [132]. PCP is now often described as the AIDS-defining illness in patients with HIV [133]. Typically, 40–75% of AIDS patients will acquire PCP at some stage during their illness [134, 135]. The use of antiviral drugs, in combination with effective prophylaxis using trimethoprim–sulfamethoxazole [133] has decreased the incidence and mortality rate, resulting in reports of a significantly higher mortality rate for immunosuppressed non-AIDS patients when compared to AIDS patients [135, 136]. Nevertheless, reports of resistance to trimethoprim, facilitated by mutations in the drugs target, have been identified in patients with PCP, raising concerns about their continued effectiveness [137].

The draft of *P. jirovecii* genome was first sequenced in 2012 from a single bronchoalveolar lavage of a patient with pneumonia [138]. However, the contiguous construction of this genome was limited due to sequence repetition, including large expansions of gene families encoding major surface glycoproteins [128]. More recently, high depth short read sequencing and long read sequencing have been utilized to improve the resolution of the *P. jirovecii* genomes [128, 129, 139]. Using comparative genomics of *Pneumocystis* spp. that infect humans and rodents, mechanisms of adaptation have been identified that allow the pathogen to thrive in mammalian hosts and evade both the innate and acquired immune defences [128]. For example, using gene-prediction tools and biochemical assays, one study has shown that *Pneumocystis* spp. lack two key fungal cell wall components: chitin and outer chain N-mannans, which may allow the organism to evade host defences [128]. Moreover, population level genome resequencing has also shown that natural populations of *P. jjrovecii* are widely distributed and display high genetic diversity, despite being associated predominantly with mammalian lung environments. This diversity may provide a selective advantage in avoiding detection of the host immune system [140].

## Current Diagnostic Methods Available

### Current Non-Molecular Clinical Diagnostics

Respiratory fungal diseases remain hard to diagnose in patients that are immunocompromised as it is often difficult to differentiate between fungal species that cause pulmonary infections through radiography [141]. For identification of invasive fungal infections, histopathology is considered one of the criteria for diagnosis [142]. However, histopathology may require the biopsy of deep tissues which exposes immunosuppressed patients to a high level of risk of infection [143]. Therefore, conventional diagnostic techniques such as culture and phenotypic analysis are regularly used to confirm *Aspergillus* spp., *Cryptococcus* spp., *Fusarium* spp., and *Scedosporeium* spp. [144] as well as to detect antifungal resistance in sputum from patients [145]. However, due to the poor sensitivity of fungal cultures, and multiple resistance phenotypes requiring multiple resistance testing, a relatively high level of mycology training is required for accurate diagnosis. Culture methods also take several days to yield any results [146] and epidemiological cut-off point, at which there is a relationship between in vitro azole susceptibility in fungal culture and clinical outcomes, have also been shown to be unclear [147]. Serum antigen assays are a more sensitive non-molecular diagnostic which can be used in conjunction with culture techniques to flag for fungal infections [146]*.* Such examples include assays for the biomarker β-d-glucan, a major component in the fungal cell wall [148] and assays for galactomannan, a polysaccharide released by *Aspergillus* spp. during growth [149]. β-D-Glucan can be found in the sera of patients infected by *Aspergillus* spp., *Fusarium* spp., and *Pneumocystis* spp., and therefore although sensitive these assays are not specific [146]. β-d-Glucan assays are also unable to detect *Mucor* or *Rhizopus* species as they do not have β-D-Glucan within their cell walls [150]. Enzyme-linked immunosorbent assays (ELISA) that target fungal-specific IgG have also been developed to detect fungal infection from patient samples [151, 152]. Although ELISA leads to quicker diagnosis than culture methods, antibody detection methods can have low specificity and associate with other closely related fungal species. Another limitation is that immunosuppressed patients, who are some of the most susceptible individuals to fungal respiratory disease, will show reduced sensitivity for these assays and do not test for antifungal resistance [153]. Therefore, as azole resistance in filamentous fungi such as *A. fumigatus* continues to emerge worldwide [154], the need for rapid and specific diagnostics using molecular-based detection becomes more apparent. Consequently, this has accelerated the development of molecular diagnostics for the detection of azole resistance in culture-negative patients.

### Current Molecular Diagnostics

When using PCR to detect *A. fumigatus* within a sample, common PCR targets that are used include the 18S rRNA gene, 28S rRNA gene and the internal transcribed spacers (ITS) [155]. Combining PCR and Sanger sequencing of *cyp51A* can detect drug resistance mutations. An advantage of using PCR over fungal culture is therefore the ability to simultaneously and quickly screen clinical samples for both *A. fumigatus* DNA and for mutations that are associated with azole resistance. This can be done by using multiplex PCRs which are able to detect multiple clinically relevant *Aspergillus* species in one assay [156]. For example, the commercially available MycoGENIE *A. fumigatus* real-time PCR kit detects the 28S rRNA gene as well as TR_34_ and L98H mutations that are associated with azole resistance within the *cyp51A* gene [157]. As well as the MycoGENIE *A. fumigatus* kit there are also several other commercial products available for the detection of AR*Af*, such as PathoNostics AsperGenius assay (PathoNostics, Maastricht, Netherlands) and Fungiplex® *Aspergillus* Azole-R in vitro diagnostics (IVD) PCR (Bruker Daltonik 75 GmbH, Bremen, Germany). However, the most recent guidelines suggest that diagnostic PCR is only used on a case-by-case basis in clinical practice. This is due to the lack of conclusive validation of commercially available assays, and the range of different methodologies currently found within literature [33]. To try and face this issue, the European *Aspergillus* PCR initiative (EAPCRI) was founded, with the aim to begin and standardize *Aspergillus* PCR methodology and validate PCR methodologies in clinical trials [158]. PCR kits have also shown poor sensitivity for detection of azole resistance directly on sputum samples and therefore still require the need for culture [159].

As is with *Aspergillus* spp., detection of fungal DNA as a means of diagnostics is a tempting approach for detection of other respiratory fungal diseases. This is especially true for infections such as *P. jirovecii* which cannot be cultured, and diagnosis is traditionally based using staining methods or fluorescent microscopy. However, these methods can often be subjective leading to low sensitivity [160]. In contrast, PCR of PCP respiratory specimens have shown as high sensitivity as 100% and can play an important role in the early diagnostics of PCP [161]. However, standard PCR cannot distinguish between airway colonization by *P. jirovecii* and pneumonia.This is a considerable challenge as sub-clinical *Pneumocystis* airway colonization occurs in up to 22% of high-risk patients and therefore the significance of a positive PCR test may be dubious [162]. This obstacle was overcome after it was found that PCP patients had a substantially higher concentrations of *Pneumocystis* DNA within Broncho Alveolar Lavage Fluid (BALF) when compared to individuals with colonization or sub-clinical infection [163]. This allows for the fungal burden to be estimated from cycle threshold values obtained through qualitative PCR in order to differentiate between pneumonia and sub-clinical infection [164]. Furthermore, commercial qPCR assays have been developed by PathNostics (Maastricht, Netherlands) to aid the detection of Pneumocystosis directly from BAL samples with high sensitivity and a sample to result in less than 3 h.

Diagnosis of mucormycosis can also benefit from PCR as Mucorales which are difficult to culture. Mucorales are also often mistaken for *A. fumigatus* in histopathology, and there are no serological tests that are commercially available [90, 165, 166]. An attractive approach is to test for Mucorales DNA in BALF or serum using PCR. Several retrospective studies, using three qPCR assays to target 18S rDNA in *rhizomucor, Mucor/Rhizopus* and *Lichtheimia* within patient serum samples, have shown Mucorales DNA can be detected on average 9 days (up to 68 days) before mucormycosis could be confirmed by culture or histopathology and up to 3 days before radiographic features were observed on CT scans [167, 168]. Currently, PCR assays cannot be used cannot be recommended as diagnosis of mucormycosis without culture and/or histological evidence [166]; however, it is hoped that with the commercial release of real-time PCR assays for the detection of clinically relevant Mucor species by PathoNostics (MucorGenius; Maastricht, Netherlands) will start to standardize assays between clinical laboratories.

PCR diagnostic techniques have also been developed to detect other fungal diseases that cause respiratory infection including *Fusarium* spp. [169], *Scedosporium* spp. [170], and *Exophiala* spp. [171]. However, analysis of larger numbers of patient samples are still needed to assess the prevalence of these assays in clinical samples before they can be accepted for regular clinical diagnostics in the near future. Nested PCR has also been designed *Cryptococcus* spp. [172] however as there are already serologic assays which have high specificity and sensitivity for *Cryptococcus* spp. [173], reducing the urgency for clinical molecular diagnostics.

## Future Directions

### Loop Mediated Isothermal AMPlification (LAMP)

PCR is often still viewed as the gold standard for quick diagnosis of fungal respiratory pathogens. However, PCR needs to be conducted in a well-equipped laboratory setting with trained personnel and can therefore not be easily deployed at a point-of-care or in an environmental setting [174]. Loop-mediated isothermal amplification (LAMP) offers an attractive alternative to PCR as it does not require temperature cycling and shows both high speed and specificity [175]. LAMP is a one-step isothermal amplification reaction which uses a DNA polymerase with a high strand displacement activity and four specific primers (two internal and two external), to produce an amplicon with a cauliflower-like structure [176]. The addition of loop primers is also able to significantly reduce the LAMP amplification time, by up to 30 min [177]. Recently, a LAMP-based assay was developed that can detect *A. fumigatus* containing the TR_34_ and TR_46_ azole resistance allele with high sensitivity, positively detecting down to 10 genomic copies/reaction in under 30 min [178, 179]. This assay was developed so it could be performed on a “lab-on-chip” platform, a diagnostic platform which uses a silicon nitride layer to bind protons released during the incorporation of nucleic acids into DNA to track DNA amplification. This data can then be sent to a smartphone device via Bluetooth which theoretically allows for easy use at a point-of-care. The application of LAMP has also been incorporated into the detection other clinically significant fungi such as *Exophiala* and *Scedosporium* species [180]. It has also been shown to be an effective point-of-care tool for Chagas disease [181] and *Salmonella* [182] and is being developed and used extensively throughout the COVID-19 pandemic to identify infections early and prevent the spread of the disease [183–185]. The only main disadvantages of LAMP are that the primers used in the assay need to be kept cold while working in the field, and even though the assay itself is easy to operate, the design of the primers themselves for detection of specific species and alleles requires expertise and detailed understanding of the target pathogen genome [174].

### Whole Genome Sequencing

Next generation sequencing technologies have allowed whole genome sequencing (WGS) to become a valuable tool within the field of mycology. WGS allows for entire genome sequencing at a cost comparable to traditional methods, such as multi-locus sequencing typing (MLST) [186]. However, MLST techniques and short tandem repeat (STR) assay methods, do still provide a fast, reproducible, inexpensive and easy method of examining population genetics of a large quantity of isolates [187]. Case in point, researchers recently analysed the genetic relatedness of 4,089 *A. fumigatus* isolates from a worldwide collection, genotyped at 9 microsatellite locations, to demonstrate, through hierarchical clustering that the species population could be divided into two clades [154]. Resistant genotypes TR_34_/L98H and TR_46_/Y121F/T289A were found to be unevenly distributed across the clades and showed to have less diverse genetic backgrounds than wild-type isolates, with identical clonal isolates being found worldwide. To allow clinicians and researchers to make informed decisions on drug administration and epidemiological infection studies this analysis has now been incorporated into a free interactive R shiny application (AfumID) to permit quick characterization of *A. fumigatus* isolates. However, WGS is becoming the method of choice for molecular epidemiological studies due to the high resolution of data it generates, at a comparatively low labour and monetary expense [188]. The application of WGS is not just limited to genotyping clinical and environmental genomes but can be used in screening for antifungal resistance, analysing of patterns of natural selection and gene flow in nature and addressing developing outbreaks of respiratory fungal diseases [189–191]. The recent development of sequencing devices such as the Oxford MinION nanopore sequencing has allowed for the rapid real-time sequencing of epidemics in resource limiting settings, which have been fully utilized in bacterial and viral outbreaks [192–194]. Real-time sequencing of fungal pathogens has also been attempted and could prove to be a promising tool to monitor future outbreaks [195]. As with other sequencing technologies, nanopore sequencing does not require previous knowledge of a pathogens genome and can be used to help construct reference genomes [196]. Coupled with its ease of portability (weighing less than 100 g) enabling quicker response times, detection of potentially unknown pathogens and can be used at point of care or in the field as demonstrated for Ebola and Zika [193, 194, 197], although it should be noted that even though the sequencing device is portable other consumables and laboratory equipment will be needed for DNA extraction. In 2018, the largest outbreak of *Candida auris* in the UK at the time was retrospectively analysed using nanopore sequencing for rapid WGS to examine the genomic epidemiology. WGS established that resistance was unlikely to have developed from multiple sources and identified isolates that had reduced susceptibility to specific antifungal drugs [191]. This was the first time that the nanopore sequencing had been used on a human fungal pathogen and the techniques illustrated here could be transferred to allow analysis of future outbreaks of human respiratory fungal pathogens. However, one of the potential difficulties that may be encountered when using nanopore for sequencing of *A. fumigatus* is that mould gDNA is prone to fragmentation [198]. Therefore, DNA extraction may first need to be optimized to in order to overcome this.

Nanopore sequencing and other WGS technologies can also be used for outbreak management, which was demonstrated in another *C. auris* outbreak within an intensive care unit (ICU) in Oxford (U.K.). Genomic analyses in this study confirmed that *C. auris* isolates on equipment within the hospital were more closely related to those collected from patients. This led to the removal of reusable temperature probes which was subsequently followed by a reduction in patient infection and control of the outbreak [199].

WGS has been increasingly used within mycology for sequencing of different *Aspergillus* species. One of its most beneficial uses is the ability to detect mutations within the *cyp51A* gene which are associated with *A. fumigatus* resistance to azoles and therefore can be used to predict drug resistance to first line therapies within a clinical setting [200]. WGS can also be used to enable high-resolution single-nucleotide polymorphisms (SNP) analysis to observe evolution and selection for antifungal resistance and potentially uncover new mechanisms of resistance [188]. Whole genome comparisons of 13 *A. fumigatus* strains with increasing azole resistance, collected from a single patient undergoing azole and echinocandin combination therapy, over a 2-year period identified 248 non-synonymous SNPs [201]. Of the isolates, nine were shown to have acquired SNPs within the *cyp51A* gene, likely as a result of azole induced pressure. However, in most of these isolates, the *cyp51A* SNPs did not fully explain the resistance profiles that were observed therefore suggests that the presence of some non-*cyp51A* SNPs are partially responsible for mediating azole tolerance through novel mechanisms [201].

### Metagenomics

The advent of next generation sequencing (NGS) has also allowed for metagenomic approaches to become an emerging option for clinical diagnosis. Unlike most molecular assays used for diagnosis of infectious disease, which only target a limited number of pathogens with specific primers/probes, approaches such as untargeted metagenomic NGS use shotgun sequencing to survey a proportion of DNA and/or RNA from within a sample without bias. Theoretically, using this method, in a single assay nearly all bacteria, fungi, parasites and viruses can be identified within a sample [202]. However, when using this method typically < 1% of reads will be non-human of which only a subset will correspond to the causative pathogen [203]. Therefore, targeted approaches can be used to increase the proportion of reads from pathogens within the sequence data. This can be done by using primers for a highly conserved region, such as 18S ribosomal RNA, *TEF1*α and internal transcribed spacer (ITS) in fungi, for universal PCR amplification and then using NGS of the amplicon to identify potential pathogens [204]. However, before metagenomic techniques can be adopted to identify fungal pathogens in clinical samples, there are several factors which cause inaccuracies that must first be overcome. These include pre-PCR biases that occur during DNA extraction due to contamination or choice of extraction kits or storage buffers and PCR biases caused by varying lengths of amplicons or primer mismatches [205]. Another limitation is the lack of a well-curated reference databases with correct taxonomic for fungal species, this can lead to misidentification and cross-talk between fungal sequences [206]. Clinical metagenomics is also exceedingly expensive to setup and could cost up to one million pounds once computer infrastructure, personnel and sequencing facilities have been considered [207].

### Communication

With new genome sequencing projects being able to produce vast quantities of sequencing data, it is crucial that genomic data is deposited in short read archives with associated metadata so that data can be synthesized across sequencing projects in order to accelerate genomic epidemiologic analysis [208]. It is also important that data can be presented in a format that will be understood by a large ranging audience [209]; This has led to the development of flexible web-browser enabled applications such as Microreact [209] which is able to display phylogeographical and temporal representation of fungal genomic analysis within the context of its own metadata, and allows for the data to be interactively visualized in the form of maps, phylogenetic trees and timelines.

## Concluding Remarks

**Fig. 2:**
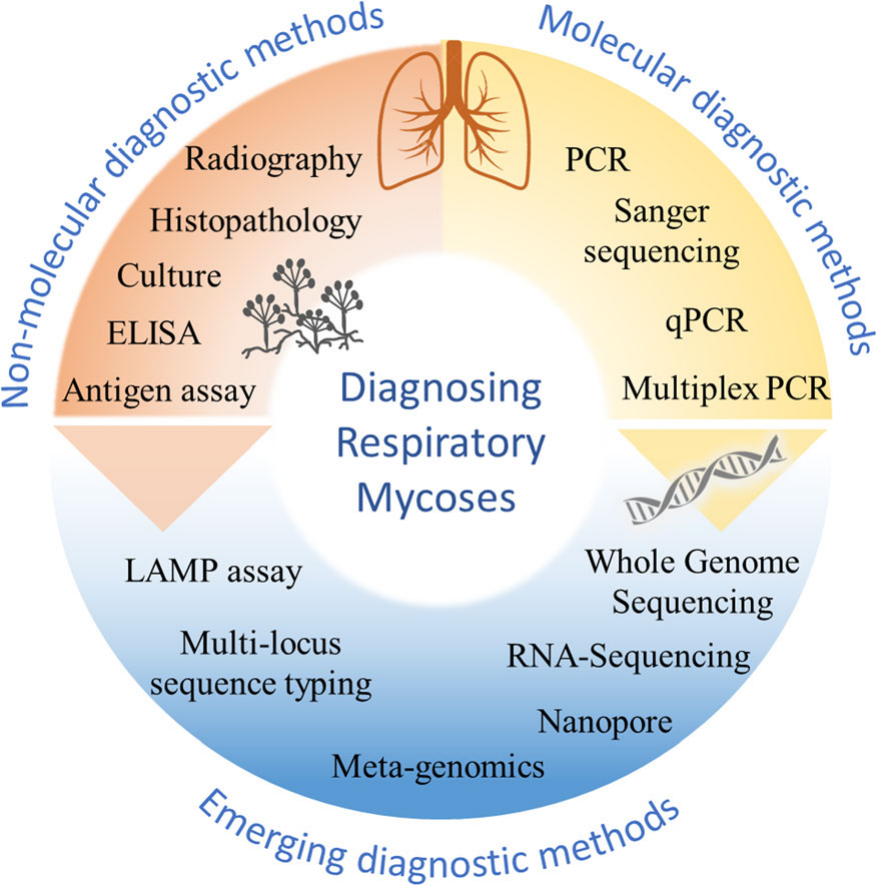
Schematic highlighting notable techniques for the diagnosis of respiratory mycoses

Significant advances in fungal genomics in recent years have undoubtably enhanced the future of nucleic acid diagnostic technologies in the field of fungal respiratory disease. Currently, conventional diagnostic techniques such as culture, microscopy and phenotypic analysis are still recommended for clinical diagnosis of fungal respiratory diseases [33]. However, molecular diagnostics and sequencing technologies offer a reproducible, cost-effective option for generating rapid data that can accelerate the epidemiology of mycoses. These technologies will increasingly be used more for medical diagnosis of fungal infections as their use within clinical laboratories increases and methodologies become standardized (Fig. 2).
